# Transoral Laser Surgery for Laryngeal Cancer

**DOI:** 10.5041/RMMJ.10146

**Published:** 2014-04-28

**Authors:** Vlad C. Sandulache, Michael E. Kupferman

**Affiliations:** 1 Bobby R. Alford Department of Otolaryngology—Head and Neck Surgery, Baylor College of Medicine, Houston, Texas, USA and; 2 Department of Head and Neck Surgery, The University of Texas M. D. Anderson Cancer Center, Houston, Texas, USA

**Keywords:** Laryngectomy, larynx, laser, swallowing, transoral, voice

## Abstract

Transoral laser microsurgery (TLM) was pioneered in the early 1970s as an approach to treat laryngeal pathology with precision and minimal thermal damage to the vocal cords. Over the last four decades, TLM has become an integral part of the treatment paradigm for patients with laryngeal cancer. TLM is one of the primary treatment options for early-stage laryngeal tumors. However, in recent years, surgeons have begun to develop TLM into a more versatile approach which can be used to address advanced laryngeal tumors. Although functional outcomes following TLM for advanced laryngeal disease are scarce, survival outcomes appear to be comparable with those reported for organ preservation strategies employing external beam radiation therapy (EBRT) and chemotherapy. In addition, TLM plays an important role in the setting of recurrent laryngeal cancer following primary irradiation. TLM has been demonstrated to decrease the need for salvage total laryngectomy resulting in improved functionality while retaining comparable oncologic outcomes. The aim of this review is to elucidate the indications, techniques, and oncological outcomes of TLM for advanced laryngeal cancers.

## HISTORY OF TRANSORAL LASER SURGERY FOR LARYNGEAL CANCER

Use of endoscopic lasers was pioneered by Strong and Jako in the early 1970s.^1^,^2^ Initially, utilization of transoral laser microsurgery (TLM) was envisioned as a treatment strategy for early laryngeal tumors, with an understanding that oncologic resection may be possible, while preserving a sufficient portion of the laryngeal framework to maintain adequate swallowing and vocalization. In 1978 Vaughan et al. described their preclinical and clinical experiences with the CO_2_ laser in the setting of laryngeal tumors.^3^,^4^ Primarily, the CO_2_ laser could be utilized either to debulk tumors, restore airway patency, or to treat smaller tumors with an oncologically sound resection. Patients were generally reported to suffer little morbidity, allowing for short hospitalizations and adequate function with regard to swallowing and voice. Importantly, the authors described the ability to avoid a tracheostomy, which is associated with substantial morbidity and cost. Davis et al. and Lacourreye et al. also described utilization of the CO_2_ laser for the purpose of debulking in the 1980s.^5^,^6^ Specifically, they suggested that partial endoscopic excision of obstructing lesions (using single or repeated treatments) can be an alternative to emergency tracheotomy or emergency laryngectomy whenever airway control can be initially ensured by endotracheal intubation.

Since the 1970s, utilization of TLM has become an important tool in the management of laryngeal tumors, and in certain centers it is considered one of the primary definitive treatment modalities for early-stage disease.

## TECHNIQUE/LIMITATIONS

Although initially designed to be used in the treatment of early laryngeal tumors in the 1970s, by the 1990s, TLM was being utilized for all tumor categories, primarily through the efforts of Steiner and colleagues.^7^–^9^ A detailed technical description of TLM is beyond the scope of this review. Authors have described a wide variety of procedures using the CO_2_ laser system, ranging from partial supraglottectomies (removal of a portion or the entire epiglottis, arytenoids, ary-epiglottic folds) to partial glottectomies to near-total laryngectomy.^10^ A detailed description of cordectomy procedures was provided in 2000 by the European Laryngology Society; these range from type I subepithelial cordectomy to type V which represent extended cordectomies encompassing either supraglottic or subglottic structures.^11^,^12^

**Figure 1.**
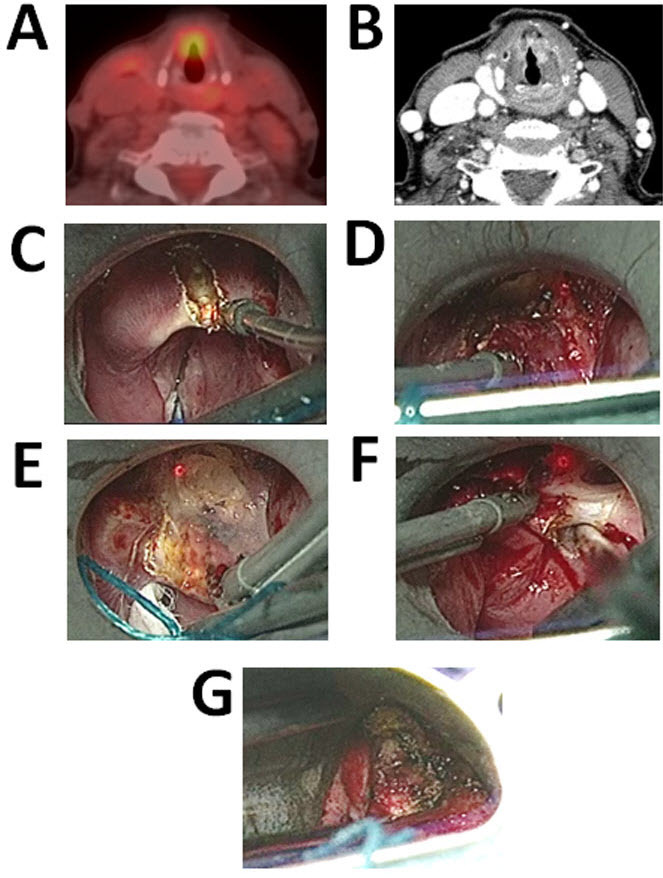
**Sixty-six-year-old Female with a History of Laryngeal Carcinoma Treated with EBRT and Chemotherapy Presents with Recurrent Laryngeal Cancer.** **A:** PET scan demonstrating FDG avid lesion of the glottis. **B:** Pre-operative CT scan demonstrating increased contrast enhancement at the level of the glottis. **C–G:** Intraoperative photographs. **C:** The epiglottis is divided in the midline. **D:** The vascular pedicle is ligated with surgical clips and divided. **E:** The dissection is carried out anteriorly into the pre-epiglottic space. **F:** The tumor is released laterally. **G:** Post partial laryngectomy evaluation of the glottis is performed to insure hemostasis.

Irrespective of the extent of surgery, TLM is based upon a number of fundamental principles that diverge substantially from traditional oncological approaches (Figure 1). First, in contrast to traditional surgical resection with en bloc tumor removal, with TLM, large tumors can be removed in a piecemeal fashion, usually as two specimens. The final tumor is then reassembled *ex vivo* for pathologic analysis of margins. Often, the epiglottis is bisected in the sagittal plane, with each hemi-larynx removed separately. In addition, since all margins are obtained using a CO_2_ laser, a pathologist trained in evaluating tissue removed via laser resection is required. As was demonstrated by Mannelli et al. in a prospective analysis of excised larynges, the thermal effect on margin status can be substantial as it relates to surgical artifact, tissue retraction, and tissue alteration. Monopolar cautery demonstrated a significantly higher degree of thermal damage compared to the harmonic scalpel or the CO_2_ laser.^13^ The authors recommended that pathologists be apprised of the surgical device type in order to adjust appropriately expectations of margin alteration. If technical limitations can be overcome, TLM confers several hypothetical advantages. Magnification and improved visualization can combine with piecemeal removal to map the tumor more accurately, reducing both the amount of normal tissue resected, and more closely following specific areas which are more infiltrative. In some ways, this resembles the approach used in Mohs resections of skin lesions.

Because the approach for TLM is endoscopic, and the skin and subcutaneous fascial envelopes are not violated, the risk of tissue breakdown and fistulization is lower. This can result in decreased utilization of pedicled or free flap reconstruction for advanced-stage tumors requiring extensive mucosal resection. It is unclear how much this particular advantage of TLM is lost when unilateral or bilateral neck dissections are required.

Canis et al. described the utilization of TLM for the removal of T4a tumors in 2013.^7^ As described by other authors, tumors were resected in multiple blocks, with margins ranging from 2–3 mm in the glottis to 5–10 mm in the supraglottis and intra-operative frozen sections being used to ascertain margin status. The operating time ranged from 2 h to 5 h depending on the extent of the tumor and the experience of the surgeon. This report suggests that there are very few technical limitations to TLM that cannot be overcome. Whether this experience is broadly applicable and, more importantly, teachable remains to be seen.

## CLINICAL OUTCOMES FOR TLM

### Early Laryngeal Cancers

Transoral laser microsurgery (TLM) represents an important tool in the management of laryngeal tumors and is commonly utilized in the treatment of early-stage disease. One current point of debate among physicians treating laryngeal tumors is whether TLM can offer similar clinical outcomes compared to organ preservation treatment consisting of external beam radiation therapy (EBRT).^14^–^18^ Feng et al. conducted a large meta-analysis comparing outcomes and costs associated with treatment of T1–T2 glottic cancers in 2011.^16^ Their analysis included 11 studies and 1,135 patients and demonstrated no significant difference in cure rates between TLM and EBRT. They were not able to conduct a substantial analysis of functional outcomes primarily because most studies to date fail to record functional outcomes using validated and reproducible scales, and only very rarely include long-term functional outcomes (see below). More recently, Lee et al. (2013) reviewed a single surgeon’s experience with TLM for 118 patients with T1–T2 glottic lesions from 1997 to 2011.^19^ At 5 years, disease-free and overall survival rates of 87.9% and 92.2% were comparable to data reported for large cohorts treated with EBRT.^20^

Taylor et al. described a multicenter cohort experience with T1b laryngeal lesions (42 patients treated with EBRT; 21 patients treated with TLM).^21^ Since involvement of the anterior commissure is often cited as a potential functional risk for patients undergoing TLM (due to anterior scarring and web formation) the data provided in this study are particularly interesting. In addition to oncologic outcomes (local control, organ preservation, disease-free survival and disease-specific survival), the authors also evaluated functional outcomes, specifically voice using the previously validated Voice Handicap Index (VHI)-10. Disease-free and overall survival at 2 years for TLM were 88.7% and 94.1%, while for EBRT they were 85.9% and 94.8%, respectively. Although vocalization data were available for less than half of all patients, no significant differences were noted between the two groups.

Agrawal et al. reported in 2007 the results from the Southwest Oncology Group (SWOG) phase II trial (single arm) evaluation TLM followed by EBRT for stage I–III supraglottic tumors.^22^ Despite its multi-institutional nature, the study only accrued 34 patients over a 4-year period. Disease-free and overall survival at 3 years were estimated at 79% and 88%, respectively. Four patients required temporary tracheostomy prior to the procedure; no patient required permanent tracheostomy; three patients were feeding tube-dependent at last follow-up. One patient required salvage laryngectomy, and two patients required salvage neck dissections. Although a significant improvement over purely retrospective series, none of these studies were randomized. Given the very disparate mechanism of treatment (EBRT versus TLM), randomized clinical trials addressing this question are unlikely in the current clinical climate.

Zhang et al. conducted an analysis in China based on 205 patients treated at a single institution with a mean follow-up of 49 months.^23^ Most tumors were glottic (70%), and most patients were reportedly N0 (78%). Approximately half of all tumors represented advanced disease (T3 20%, T4 25%). Surgical treatment of primary lesions consisted of total laryngectomy (*n*=71), partial laryngectomy or TLM (*n*=134). TLM or open partial laryngectomy was reserved for patients with T stage less than T3 and was performed routinely only after 2000. No individual survival or functional data were provided for patients treated with TLM, but the study does demonstrate propagation of the technique outside of the initial centers that developed it in the 1970s and 1980s. Pukander et al. similarly reported the Finnish experience with TLM across all stages of laryngeal cancer in 2001.^24^ Following initiation of TLM as a clinical treatment option, the authors were able to treat 140 patients within a 4-year span. Survival and recurrence data were detailed only for early-stage disease. Based on the current literature, it is clear that TLM is increasingly becoming part of the treatment paradigm for laryngeal tumors throughout the world and represents an alternative to definitive EBRT that offers equivalent local control and functional outcomes.

### Advanced Laryngeal Cancer

In recent years, an increasing number of centers have reported experience with TLM in advanced laryngeal disease (Table 1).^8^,^16^,^25^–^28^ Although data are primarily obtained from retrospective patient cohorts, there appear to be significant data to support utilization of TLM in the setting of advanced laryngeal cancer. Although Pukander and Zhang reported the outcomes for patients with advanced disease as part of larger cohorts, Vilaseca et al. evaluated outcomes in 147 patients with T3 laryngeal tumors following TLM treatment.^29^ Overall survival in this patient group at 5 years was 53%. Neck dissection was performed in 66% of patients, and 25% of patients required adjuvant irradiation of the primary site, while 12% required irradiation of the neck. Over one-third of patients experienced local recurrence which required additional TLM, open partial laryngectomy, and salvage total laryngectomy in 9%, 9%, and 81.8% of patients, respectively.

**Table 1.** Clinical Outcomes for Advanced Laryngeal Cancer Treated with TLM.

**Author**	**Year**	***n***	**Site**	**EBRT +/− Chemotherapy (*n*)**	**LC (%)**	**OS (%)**
Iro^26^	1998	141	SG	63	III—75% 5 y IV—78% 5 y	NA
Hinni^25^	2007	117	SG, G	45	68% 5 y	55% 5 y
Vilaseca^29^	2010	147	SG, G	36	NA	53% 5 y
Canis^8^	2013	226	SG, G (T3)	40	72%	64% 5 y
Canis^7^	2013	79	SG, G (T4)	31	67%	56% 5 y

EBRT, external beam radiation (used in the adjuvant setting); G, glottis; LC, locoregional control; OS, overall survival; SG, supraglottis.

More recently, Canis et al. also analyzed outcomes for patients with advanced disease stage (T3) treated with TLM.^8^ Tumors were relatively evenly divided into glottic and supraglottic (54% versus 46%). Patients were treated by TLM with (63%) or without selective neck dissection. Eighteen percent of patients required postoperative EBRT, which is not surprising given the stage of the primary tumors and the percent of tumors which were supraglottic in origin. Disease-free and overall survival at 5 years were 63% and 64.4%, respectively. Complications related to treatment included six temporary tracheostomy tubes, two permanent tracheostomy tubes, and three permanent gastrostomy tubes. It is important to note that although this is by far the largest cohort of patients treated with TLM for advanced disease published to date, it spans a period from 1980 to 2006. Since treatment was provided by a group led by one of the developers of TLM (Steiner), these data may represent the very best of what can be expected using this treatment paradigm. These data are largely consistent with data reported earlier in 1998 by Iro et al. which demonstrated disease-free survival at 5 years of 76% for stage III disease treated with surgery alone and 69% for disease treated with surgery and adjuvant radiation; disease-free survival for stage IV disease treated with surgery alone versus surgery and adjuvant radiation was 100% and 49%, respectively.^26^

The analysis of T3 tumors was extended in a parallel manuscript by this group.^7^ The authors conducted a retrospective analysis of 79 patients with previously untreated T4a laryngeal tumors (39% glottic, 61% supraglottic). Consistent with the advanced stage of the primary tumor, 43 patients required unilateral or bilateral neck dissections. Adjuvant EBRT with or without chemotherapy was utilized in 31 patients. The rate of laryngeal preservation at 5 years was 80%. The 5-year overall survival rates were 62.5% in patients without cervical metastasis and 57.2% in patients with cervical metastasis. Disease-free survival at 5 years was 61.9%. Thirteen patients required a temporary tracheostomy, and two patients required a total laryngectomy secondary to persistent laryngeal dysfunction. Only four patients required a permanent gastrostomy tube placement, but 62 patients required temporary nasogastric feeding. No information was provided on vocalization and long-term swallowing function measurements. The low rate of salvage laryngectomy or permanent gastrostomy is very encouraging considering the advanced T stage of the tumors evaluated in this study. The median follow-up of 49 months should have been sufficient to detect persistent laryngeal dysfunction in the postoperative period, yet an overwhelming percentage of these patients appear to have recovered sufficient function postoperatively to maintain adequate swallowing.

Although the Canis et al. studies are quite encouraging with regard to clinical outcomes for advanced disease, they represent the work of a group with very extensive experience in TLM and may not be reproducible in other settings. In 2007 Hinni et al. reported data on 117 patients with stage III–IV laryngeal disease.^25^ This analysis is important because it represented the combined experience of surgeons at the Mayo Clinics in Scottsdale and Jacksonville, Washington University and the University Hospital in Gottingen (prospectively collected data for patients with advanced disease treated between 1997 and 2004). Of these patients, 91 underwent neck dissection and 45 required postoperative radiotherapy. In this patient cohort, organ preservation at 2 and 5 years was 92% and 86%, while 2-year disease-free survival and overall survival were 68% and 75%, respectively. Complications included permanent supraglottic stenosis in two patients, persistent tracheostomy dependence in two patients, and persistent feeding tube dependence in four patients (secondary to aspiration). Of note the authors recorded four treatment-related deaths (3%). Within this patient cohort, the complication rate appears to be higher compared to the Canis et al. studies.

Use of TLM as a primary treatment modality for advanced laryngeal tumors is likely to remain controversial in the near future. In the absence of level I data demonstrating equivalence for T3 disease TLM is unlikely to replace chemo-EBRT as the primary treatment paradigm. Nevertheless, data from the above studies are encouraging when compared to data from chemo-EBRT trials such as RTOG 91-11. Within the scope of 91-11, treatment-related toxicity was substantial (60%–80%).^30^ As reported in a recent update, laryngeal preservation across the three treatment arms at 5 years ranged from 66% to 84%, and from 64% to 82% at 10 years. Disease-free and overall survival at 5 years ranged from 28% to 38%, and from 54% to 58%, respectively, across the treatment arms.^31^ It is also important to note that, in the setting T3 stage laryngeal tumors, a significant percentage of patients will require adjuvant radiation, and in certain cases adjuvant chemotherapy. In these patients, the benefit of TLM remains unclear since their organ is not spared radiation. There are currently no data to suggest that TLM followed by radiation provides superior oncologic outcomes to definitive EBRT alone.

Whether TLM can replace open laryngectomy for large T3 or T4 tumors remains to be seen and is likely to be a function of how easily TLM skills can be conferred to trainees. Vilaseca and colleagues evaluated outcome data from 587 patients treated by five surgeons between 1998 and 2012.^32^ Their data indicate that more experienced surgeons required fewer interventions to achieve oncologic cure and performed fewer salvage laryngectomies following TLM. The rate of complications as well as positive margins did not differ between the surgeons. Subset analysis of locally advanced tumors, however, revealed that surgeon experience had a significant impact on the number of surgeries required for each patient, overall complication rate, and disease-free survival.

Open resection of large laryngeal/pharyngeal tumors often requires reconstruction with pedicled or free flaps, particularly in the setting of previously irradiated tissue. Since TLM does not violate the skin and fascial planes, the risk of salivary leak/fistulae and the need for extensive reconstruction following oncologic ablation are reduced.

### Recurrent Laryngeal Cancer

Given the increase in organ preservation strategies (EBRT versus chemo-EBRT) for treatment of laryngeal tumors, a significant proportion of surgical treatment currently occurs in the salvage setting. This is in part driven by the propensity of laryngeal squamous cell carcinoma (SCC) to develop through a field cancerization phenomenon driven by generalized exposure to conventional carcinogens.^33^

As discussed above, non-surgical treatment of early glottic tumors represents the primary treatment paradigm, at least in the United States.^34^,^35^ Although cure rates are extremely high, patients with laryngeal cancer exhibit significant rates of recurrence (early or late) as well as second primary tumor development. Since most patients cannot be re-irradiated to a curative dose, treatment for recurrent laryngeal cancer is primarily surgical. Within the context of recurrent laryngeal tumors, TLM has gained increased recognition as a useful treatment paradigm (Table 2). Two primary themes are evident from existing literature on TLM for recurrent disease. First, the rate of complications is higher than in the primary treatment setting. This is not surprising, as radiated tissue has been found to heal much more slowly, and patients with recurrent disease are generally in a more frail overall state. Second, in the recurrent setting, multiple subsequent procedures can be, and often are, used to achieve local control. Utilization of total laryngectomy is often the ultimate salvage option once more conservative surgical approaches have failed.

**Table 2.** Clinical Outcomes for Recurrent Laryngeal Cancer Treated with TLM.

**Author**	**Year**	***n***	**TL (*n*)**	**OS (%)**
Reynolds^37^	2013	16	NA	50% (30 mo)
Hong^38^	2013	7	1	68.6%
Del Bon^39^	2012	35	4	91% (5 y)
Roedel^40^	2010	53	14	53% (5 y)
Kerrebjin^41^	1992	23	8	NA
Steiner^9^	2004	34	6	53 (5 y)

OS, overall survival; TL, patients requiring total laryngectomy as the final salvage procedure.

Ramakrishnan and colleagues conducted a meta-analysis of 11 previously published studies on TLM for recurrent laryngeal cancer following primary EBRT or chemo-EBRT.^36^ Their analysis demonstrated disease-free survival at 2 years to be 70.9% (174 patients) and overall survival to be 74.8% (276 patients) with a 72.3% (286 patients) rate of laryngeal preservation. A majority (91.5%) of patients presented with early-stage recurrent disease (Tis-T2). A significant proportion of patients required multiple interventions in order to achieve oncologic cure. Local control increased from 56.9% following a first TLM procedure to 63.8% following repeat intervention. Results from this meta-analysis are consistent with smaller individual studies detailed below.

Reynolds et al. reported data acquired over an 8-year period on 16 patients with recurrent laryngeal and oropharyngeal tumors.^37^ Disease-free and overall survival were 68.8% and 50%, respectively, with a mean follow-up of almost 30 months. The authors noted a significant rate of complications which represents a departure from studies reporting TLM use in the setting of a previously untreated tumor. These findings are consistent with those of Hong et al.^38^ Over a 4-year period, seven patients with tumors ranging from T1 to T3 were treated with TLM with or without neck dissection for recurrent laryngeal cancer. The reported local control rate was 100%, although laryngeal preservation could be achieved in only 86% of patients. One patient which recurred at 8 months following TLM required salvage total laryngectomy. Del Bon et al. reviewed 35 patients treated between 1995 and 2009.^39^ The patients presented with tumors ranging from T1a (*n*=16) to T3 (*n*=2). Overall survival at 5 years was 91%, while laryngeal preservation was 87%, similar to the Hong et al. and higher than the Reynolds et al. studies.^37^,^38^ Roedel and colleagues evaluated clinical outcomes in 53 patients treated with TLM for recurrent laryngeal tumors following EBRT with a mean follow-up of 88 months.^40^ Patients included both early and advanced disease (T3–4). Approximately half (42%) achieved cure using a single TLM procedure, while 31 patients developed a second recurrence following TLM. Of these, 10 underwent successful repeat TLM. In the remaining 20 patients, salvage laryngectomy was required in 14 patients, while 6 were slated to palliative treatment. Overall survival at 5 years was 53.3%, while disease-free survival was 68.6%. Of note, recurrence following first TLM was associated with a significant decrease in both overall and disease-free survival. The use of multiple procedures, either endoscopic or open, to achieve cure in this setting was also described by Kerrebjin et al.^41^ Of 23 patients with recurrent glottis SCC following EBRT, 15 patients were cured with a single TLM procedure, while 8 patients required total laryngectomy for repeated post-TLM recurrence.

A recent review by Motamed et al. focusing on larger patient cohorts identified local control rates for early recurrent disease of 77% and 65% for open versus TLM approaches.^42^ When salvage total laryngectomy was added, local control rates reached 90% and 83%, respectively. Steiner et al. also reported that a significant percentage of patients required additional surgery to achieve local control following recurrence.^9^ Of 34 patients, 71% were cured with a single TLM procedure, while 6 patients required total laryngectomy, and 3 patients were slated for palliative treatment. Salvage treatment resulted in disease-free and overall survival of 86% and 53%, respectively, at 5 years.

Although the above studies clearly demonstrate utility for TLM in the setting of recurrent laryngeal SCC, several questions remain unanswered. First, are outcomes the same for residual disease, recurrent disease, or true second primary tumors? Second, are TLM procedures associated with more or fewer treatment-related complications compared to open partial laryngectomy procedures? Third, how is survival (disease-free and overall) impacted by the need for multiple procedures (only one article from those listed above addresses this question)?

## FUNCTIONAL OUTCOMES FOLLOWING TLM

Given the absence of randomized, prospective trials involving TLM, there is a scarcity of level I evidence on functional outcomes following TLM treatment of laryngeal tumors. Very few authors have compared functional outcomes between patients treated with TLM compared to patients treated with EBRT +/− chemotherapy, and most of the existing studies involve early-stage tumors. Kerr et al. compared voice outcomes following treatment for early glottic tumors across three academic centers.^43^ Laryngeal preservation at 2 years was comparable between TLM and EBRT, but Voice Handicap Index (VHI) scores were lower from TLM-treated patients between 6 and 48 months post treatment. Vilaseca et al. reported data from a prospective longitudinal study involving 93 patients treated with TLM.^28^ Overall quality of life ascertained using the previously validated University of Washington Quality of Life Questionnaire (UW-QOL) tool demonstrated improvement from pre-treatment status following TLM, as did voice. Adjuvant radiation and neck dissection were negatively associated with QOL measures; advanced tumors resulted in decreased quality of life. These findings are similar to those of Robertson et al.^44^ Questionnaires including the Voice Symptom Scale (VoiSS), MD Anderson Dysphagia Inventory (MDADI), and UW-QOL were used to analyze functional outcomes for 147 patients. Most patients presented with early disease (47% T1, 35% T2). The authors found no difference in QOL between patients with T1 disease treated with TLM (*n*=43) and those treated with EBRT (*n*=26). However, swallowing (measured using MDADI) and voice (measured using VoiSS) as well as overall QOL (measured using UW-QOL) were significantly worse in patients with more advanced T stage at presentation. Unfortunately, since functional data were not available prior to treatment, it is unclear whether the effects of disease stage on ultimate function are driven by tumor destruction of normal tissue or by the treatment itself. Hirano et al. partially addressed this issue through a detailed study of vocal fold function in patients treated with TLM (*n*=17) and patients treated with definitive EBRT (*n*=14) for T1a laryngeal lesions.^45^ This analysis demonstrated decreased voice quality in TLM patients, associated with impaired vibration and incomplete glottal closure as determined using stroboscopy.

Definitive conclusions regarding functional outcomes following TLM are limited by the available data in the literature. Van Loon and colleagues conducted a systematic review of functional outcomes following treatment for laryngeal cancer and arrived at a similar conclusion.^27^ The authors concluded that standardization of QOL measurements as well as precise descriptions of tumor size and depth is required to allow for appropriate comparisons across treatment groups. A systematic review by Spielmann et al. in 2010 was similarly unable to reach definitive conclusions.^46^ Whether this can be accomplished in the near future remains to be seen.

## CONCLUSION

Over the last four decades, TLM has evolved from a tool used for excision of small primary tumors and debulking into a robust surgical treatment modality that can be used to tackle a wide range of laryngeal tumors. TLM currently plays an important role in the setting of advanced laryngeal cancer and, with additional technical development, may begin to replace traditional partial laryngectomy techniques. In the setting of recurrent laryngeal cancer, TLM provides a useful means of surgical salvage which can substantially prolong laryngectomy-free survival resulting in improved patient quality of life.

## Abbreviations:

EBRT: external beam radiation therapy
MDADI: MD Anderson Dysphagia Inventory
QOL: quality of life
SWOG: Southwest Oncology Group
SCC: squamous cell carcinoma
TLM: transoral laser microsurgery
UW-QOL: University of Washington Quality of Life Questionnaire
VHI: Voice Handicap Index
VoiSS: Voice Symptom Scale.
